# Aldose reductase mediates endothelial cell dysfunction induced by high uric acid concentrations

**DOI:** 10.1186/s12964-016-0158-6

**Published:** 2017-01-05

**Authors:** Zhiyong Huang, Quan Hong, Xueguang Zhang, Wenzhen Xiao, Liyuan Wang, Shaoyuan Cui, Zhe Feng, Yang Lv, Guangyan Cai, Xiangmei Chen, Di Wu

**Affiliations:** 1 Department of Nephrology, Chinese PLA General Hospital, Chinese PLA Institute of Nephrology, State Key Laboratory of Kidney Diseases, National Clinical Research Center of Kidney Diseases, Beijing, 100853 People’s Republic of China; 2 Department of Nephrology, The 175th Hospital of PLA, Zhangzhou Fujian, 36300 People’s Republic of China; 3 Division of Nephrology, Department of Medicine, Icahn School of Medicine at Mount Sinai, New York, NY 10029 USA

**Keywords:** Aldose reductase, Endothelial cell dysfunction, Uric acid, Reactive oxygen species, Hyperuricemia, CKD

## Abstract

**Background:**

Uric acid (UA) is an antioxidant found in human serum. However, high UA levels may also have pro-oxidant functions. According to previous research, aldose reductase (AR) plays a vital role in the oxidative stress-related complications of diabetes. We sought to determine the mechanism by which UA becomes deleterious at high concentrations as well as the effect of AR in this process.

**Method:**

Endothelial cells were divided into three groups cultured without UA or with 300 μM or 600 μM UA. The levels of total reactive oxygen species (ROS), of four ROS components, and of NO and NOX4 expression were measured. Changes in the above molecules were detected upon inhibiting NOX4 or AR, and serum H_2_O_2_ and vWF levels were measured in vivo.

**Results:**

Increased AR expression in high UA-treated endothelial cells enhanced ROS production by activating NADPH oxidase. These effects were blocked by the AR inhibitor epalrestat. 300 μM UA decreased the levels of the three major reactive oxygen species (ROS) components: O_2_•-, •OH, and ^1^O_2_. However, when the UA concentration was increased, both O_2_•- levels and downstream H_2_O_2_ production significantly increased. Finally, an AR inhibitor reduced H_2_O_2_ production in hyperuricemic mice and protected endothelial cell function.

**Conclusions:**

Our findings indicate that inhibiting AR or degrading H_2_O_2_ could protect endothelial function and maintain the antioxidant activities of UA. These findings provide new insight into the role of UA in chronic kidney disease.

**Electronic supplementary material:**

The online version of this article (doi:10.1186/s12964-016-0158-6) contains supplementary material, which is available to authorized users.

## Background

Uric acid (UA) is the final enzymatic product in the degradation of purine nucleosides and free bases in humans and the great apes [1–3]. UA is a powerful antioxidant that scavenges singlet oxygen (^1^O_2_) molecules, oxygen radicals, and peroxynitrite (ONOO^−^) molecules. UA also chelates transition metals to reduce ion–mediated ascorbic acid oxidation [4–7]. UA is responsible for approximately 50% of serum antioxidant activity [2, 4]. However, in vivo and cellular studies have demonstrated that depending on its chemical microenvironment, UA may also be a pro-oxidant [8]. Strong epidemiological evidence suggests that the prevalence of gout and hyperuricemia is increasing worldwide [9]. High UA levels are strongly associated with and often predict the development of hypertension, visceral obesity, insulin resistance, dyslipidemia, type II diabetes, kidney disease, and cardiovascular events [10, 11]. Although endothelial dysfunction generally occurs in the initial stages of these diseases, few studies on the effect of UA on human endothelial cells have been performed [12]. UA dose-dependently decreases nitric oxide (NO) production in intact bovine aortic endothelial cells [13], and hyperuricemia induces endothelial dysfunction via mitochondrial Na^+^/Ca^2+^ exchange-mediated mitochondrial calcium overload [14]. However, how the effects of UA become deleterious at high concentrations is unknown. Although the pathogenesis of these diseases is extremely complex and incompletely understood, oxidative stress clearly plays a central role. The urate oxidant-antioxidant paradox led us to investigate the point at which urate becomes an oxidant and the pathway through which this occurs. Although UA may have protective effects under certain conditions [15, 16], it cannot scavenge all radicals. Additionally, UA and/or its downstream radicals can trigger intracellular oxidant production via the ubiquitous NADPH oxidase-dependent pathway, thereby resulting in oxidative stress.

Aldose reductase (AR) is the rate-limiting enzyme in the polyol pathway, and NADPH acts as a cofactor [17]. AR plays an important role in the pathogenesis of diabetic complications [18] and atherosclerosis [19]. In diabetic rats, AR expression was increased, and inhibiting AR ameliorated renal function [20]. The AR inhibitor epalrestat suppresses the progression of diabetic complications such as retinopathy, nephropathy and neuropathy [21]. Genetic AR deficiency also prevented the progress of diabetic nephropathy [22]. The elevation of AR may be related to higher oxidative stress levels in diabetic rats [23]. In our previous research, AR expression was increased in endothelial cells treated with high uric acid concentrations [24]. Increased substrate flux via AR leads to increased ROS production, cell injury, apoptosis, altered ion regulation, and mitochondrial dysfunction [25–31], and increased ROS production is associated with NADPH oxidase activation [32–34].

In this study, we detected the different ROS types generated in HUVECs cultured with different UA concentrations as well as the changes in ROS production upon transfecting HUVECs with siRNA against AR. We then investigated the role of AR in UA-induced endothelial injury in vitro and in vivo. Our results suggest a novel mechanism underlying the endothelial dysfunction caused by high UA levels.

## Methods

### Cell culture and uric acid preparation

Human umbilical vein endothelial cells (HUVECs) were purchased from YRbio (Cat#NC006, Changsha, China) and cultured in RPMI-1640 media supplemented with 10% fetal bovine serum (FBS) at 37 °C in a humidified incubator in a 5% CO_2_ atmosphere.

Uric acid was purchased from Sigma–Aldrich (Carlsbad, CA). Uric acid powder was dissolved in a 1 mol/L NaOH solution at a concentration of 40 mmol/L. Then, the uric acid solution was added to the serum containing medium at a final concentration and at a pH 7.2–7.4.

### Intracellular reactive oxygen species (ROS) assays

Cells were seeded onto 35-mm confocal dishes (with a cover glass) and classified into control, normal uric acid (UA), and high uric acid (HUA) groups (*n* = 3). Cells in the UA and HUA groups were cultured in medium containing 300 or 600 μM uric acid, respectively, for 24 h followed by incubation with the total oxidative stress indicator chloromethyl derivative dichlorodihydrofluorescein diacetate (CM-H_2_DCFDA, Beyotime, Nanjing China, 5 μM) for 30 min in the dark at 37 °C. After three washes in PBS, green fluorescence was visualized using a laser scanning confocal microscope at an excitation wavelength of 488 nm and an emission wavelength of 515 nm.

Before HUA treatment, the HUA group was pretreated with 0.5 mM apocynin (Santa Cruz Biotechnology) or 0.1 μM epalrestat (Santa Cruz Biotechnology). Pyocyanin (100 μM; Sigma) was used as a positive control in the comparisons of ROS production.

### Detection of intracellular ROS components

For the total intracellular ROS levels, method was referenced previously by the oxidant-sensing fluorescent probe CM-H 2 DCFDA [14]. Briefly, this probe was loaded into previously subcultured HUVEC-Cs at a final concentration of 10 μmol/L, and the cells were then cultured for 30 min at 37 °C. After incubation, the culture medium was washed twice with PBS and analyzed by laser confocal microscopy with an excitation wavelength of 488 nm and an emission wavelength of 515 nm.

The detection of intracellular ROS components occurred as follows. For O_2_^•−^(Mitochondrial): cells were incubated with 4 μmol/L Mito-SOX Red (Invitrogen) in the dark at 37 °C for 10 min, and red fluorescence was observed at an excitation wavelength of 510 nm and an emission wavelength of 580 nm. For H_2_O_2_: cells were incubated with 30 μmol/L BES-H_2_O_2_ (Seebio, China) in the dark at 37 °C for 1 h, and green fluorescence was observed at an excitation wavelength of 485 nm and an emission wavelength of 515 nm. For · OH: cells were incubated with 100 μmol/L proxylfluorescamine (Invitrogen) in the dark at 37 °C for 30 min, and green fluorescence was observed at an excitation wavelength of 488 nm and an emission wavelength of 520 nm. For ^1^O_2_: cells were incubated with 20 μmol/L trans-1-(2′-methoxyvinyl)pyrene (Invitrogen) in the dark at 37 °C for 10 min, and blue fluorescence was observed at an excitation wavelength of 405 nm and an emission wavelength of 460 nm. For ONOO^−^: cells were incubated with 10 μmol/L dihydrorhodamine123 (Santa Cruz) in the dark at 37 °C for 30 min, and green fluorescence was observed at an excitation wavelength of 488 nm and an emission wavelength of 520 nm.

### Generation of inducible and stable cell lines

Reverse transcription was carried out on human kidney RNA with Superscript II reverse transcriptase, according to the manufacturer’s instructions. The full-length human NOX4 was amplified by PCR using the primers NOX4_F, 5′-GGGGACAAGTTTGTACAAAAAAGCAGGCTTCACCATGGCTGTGTCCTGGAGG-3′, and NOX4_R, 5′-GGGGACCACTTTGTACAAGAAAGCTGGGTCTCA GCTGAAAGACTCTTTATTGTATTC-3′. The PCR product was cloned into a pcDNA3.1 vector according to the manufacturer’s instructions, obtained pcDNA-NOX4. HUVECs were transfected with the pcDNA-NOX4 plasmid using Lipofectamine 2000 (Invitrogen). Clones were selected 10–16 days after transfection using 400 μg/ml neomycin (G418) to obtain HUVECs stably expressing NOX4.

### Measurement of nitric oxide levels in culture supernatants or serum

Before treatment with chemicals or UA, media were replaced with Dulbecco’s modified Eagle’s medium (DMEM). The supernatant or serum was centrifuged and subjected to NO level evaluation using the Nitric Oxide Assay Kit (Applygen Technologies, China) according to the manufacturer’s instructions. The end-point measured formula was NO_2_^−^_._.

### Real-time PCR

RNA was extracted from tissues and cells using TRIzol reagent (Invitrogen) and reverse transcribed into cDNA using M-MLV reverse transcriptase (Invitrogen). The cDNA was used as a template in quantitative real-time PCR reactions performed using SYBR green I PCR Master Mix and an ICycler system (Bio-Rad). The following primers were designed from the full-length AR and *Nox4* mRNA sequences and synthesized by SBS Biotechnology Corporation (Beijing, China): AR sense, 5′- CCTATGGCCAAGGACACACT-3′ and antisense, 5′-CTGGTCTCAGGCAAGGAAAG-3′; NOX4 sense, 5′-TTGCCTGGAAGAACCCAAGT -3′ and antisense, 5′- TCCGCACAATAAAGGCACAA-3′. As an internal control, mouse GAPDH was amplified using the following primers: sense, 5′-GGCATGGACTGTGGTCATGAG-3′ and antisense, 5′-TGCACCACCAACTGCTTAGC-3′. Relative expression (fold change vs. control) was quantified using the 2^-ΔΔCt^ method.

### Western blotting

For Western blotting, proteins were extracted from tissues or cells using RIPA lysis buffer (50 mM Tris-HCl, pH 7.5, 150 mM NaCl, 0.5% deoxycholate, 1% Nonidet P-40, 0.1% SDS, 1 mM PMSF, and protease cocktail at 1 μg/ml). Protein concentrations were measured using a BCA kit (Pierce). Protein samples (60 μg per lane) were separated by 12% SDS-PAGE and transferred to nitrocellulose (NC) membranes. After staining with Ponceau S, the membranes were incubated overnight at 4 °C in 5% non-fat milk followed by incubation with a primary antibody against AR (Santa Cruz Biotechnology) or β-actin (Sigma). Immunoreactive bands were visualized using ECL reagent (Santa Cruz Biotechnology) according to the manufacturer’s instructions and were then exposed to X-ray film. Protein band intensities were quantified using the Quantity One software (Bio-Rad). The assay was repeat 3 times.

### Aldose reductase activity assays

AR activity was measured spectrophotometrically as previously described [35, 36]. Briefly, AR activity was measured as the decrease in the absorbance of NADPH at 340 nm using DL-glyceraldehyde as the substrate. The assay mixture contained 30 mM potassium phosphate buffer (pH 6.5), 5 mM DL-glyceraldehyde, 0.2 M ammonium sulfate, and 1.0 mM NADPH. The results are presented as μmol NADPH · min-1 · g-1 protein. All reagents were from Sigma. The assay was repeat 3 times.

### Establishment of hyperuricemic mouse models

Hyperuricemic mouse models were established as described by Yang et al. [37] with slight modifications. The animal protocol was reviewed and approved by the Institutional Animal Care and Use Committee of the Chinese PLA General Hospital. Wild-type C57BL/6 mice obtained from the Experimental Animal Center of the Academy of Military Medical Sciences (China) were used as controls. The mice were housed in temperature-controlled cages on a 12-h light-dark cycle and given free access to water and normal chow. After one week of breeding for adaptation, the mice were grouped into control (*n* = 6) and hyperuricemic model (*n* = 24) groups. Mice were intraperitoneally injected with 250 mg/Kg · d oxonic acid potassium salt (Sigma) and 250 mg/Kg · d uric acid (Sigma). After receiving intraperitoneal injections for 3 days, the hyperuricemic model group was sub-classified into hyperuricemic mice (*n* = 6), hyperuricemic mice treated with epalrestat (100 mg/Kg · d, Dyne, China) (*n* = 6), and hyperuricemic mice treated with polyethylene glycol catalase (PEG-catalase, 12000 U/Kg · d, Sigma) (*n* = 6). The antioxidant treatments PEG-catalase and epalrestat were intragastrically administered. After 10 days of modeling, the levels of UA, NO, H_2_O_2_, and von Willebrand factor (vWF) in the blood were evaluated.

### Measurement of serum UA, H_2_O_2_ and vWF levels

The serum UA level was assayed using an enzymatic method that measures the end production of quinonimine using an automatic biochemical analyzer (Hitachi, Japan). Serum H_2_O_2_ levels were assayed using a hydrogen peroxide assay kit (NJJCbio, Nanjing, China) with the end formula Mn^2+^. vWF was detected using a von Willebrand Factor ELISA kit.

### Statistical analyses

All data are expressed as the means ± SD. Mean comparisons among multiple groups were conducted using one-way analysis of variance (ANOVA). Comparisons of the means between two groups were conducted using randomized controlled *t*-tests. A *p* value < 0.05 was considered statistically significant.

## Results

### High UA increased intracellular ROS production, AR activity and endothelial cell impairment but decreased NO release

**Fig. 1:**
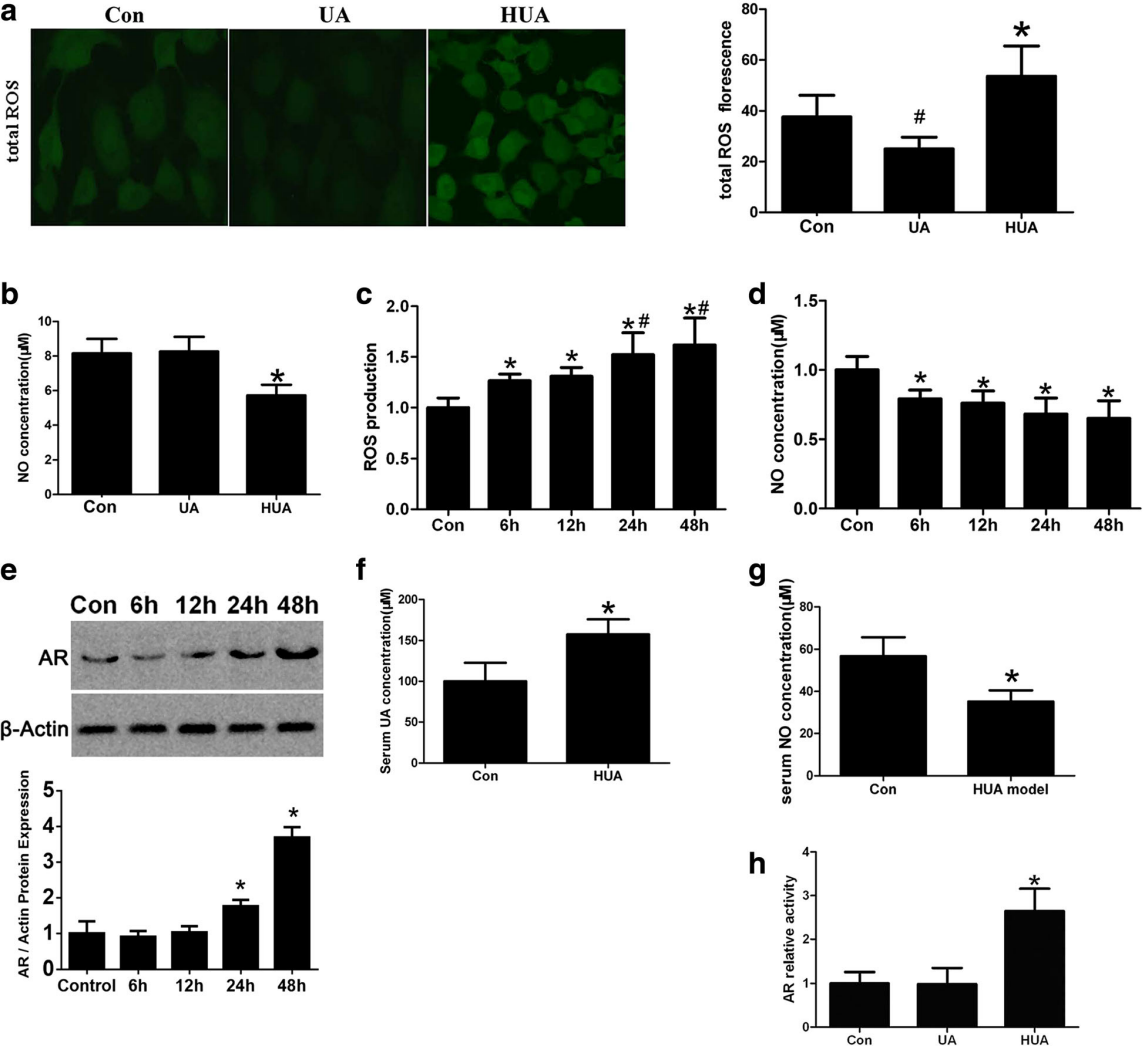
Effect of different UA concentration on intracellular oxidative stress, NO release and AR activity. Endothelial cells were cultured in the presence of 300, 600 μM UA or without UA for 24 h. **a** Total ROS was reduced in cells treated with 300 μM UA (^#^ *P <* 0.05 vs. control, *n* = 3), but increased in cells treated with 600 μM UA (*P < 0.05 vs. control, *n* = 3). **b** Compared to the control, the NO level was not significantly different in the 300 μM UA-treated group, but was reduced in the 600 μM UA-treated group (*P < 0.05 vs. control, *n* = 6). **c** Cells were treated with 600 μM UA at 6, 12, 24, and 48 h; total ROS production increased (*P < 0.05 vs. control, *n* = 6), ROS production was saturated at 24–48 h (#*P <* 0.05 vs. 6 h or 12 h, *n* = 6), and (**d**) NO levels decreased at 6 h (*P < 0.05 vs. control, *n* = 6). **e** AR expression increase at 24 h (*P < 0.05 vs. control, *n* = 3) in the high concentration of uric acid. There is no significate change at the 6 h or 12 h. **f, g** After intraperitoneal injection with oxonic acid potassium salt and UA for 10 days, serum UA levels in wild-type C57BL/6 mice increased significantly (*P < 0.05 vs. control, *n* = 6), whereas serum NO levels declined (*P < 0.05 vs. control, *n* = 6). **g** AR activity increased in endothelial cells cultured in the presence of 600 μM UA for 24 h, but there was no change upon treatment with 300 μM UA (*P < 0.05 vs. control, *n* = 6)

To confirm the impairment of endothelial cells by UA treatment, we evaluated the effect of different UA concentrations on ROS production and NO release in HUVECs UA (300 μM) reduced total ROS levels in endothelial cells, whereas high UA (600 μM) treatment increased intracellular ROS production (Fig. 1a). NO release was reduced after high UA treatment in vitro with the turning point of 500 μmol/L, An additional file shows this in more detail [see Additional file 1] but unchanged after UA treatment (Fig. 1b). Additionally, total ROS production increased and NO levels decreased in a time-dependent manner in cells treated with high UA, AR protein expression increased at 24 h and 48 h of high concentration uric acid treated (Fig. 1c, d and e). In male hyperuricemic C57BL/6 mice after modeling in vivo serum UA levels significantly increased (Fig. 1f), whereas NO levels decreased (Fig. 1g). Furthermore, AR activity increased in endothelial cells (Fig. 1h). Our results showed that AR activity increased upon treatment with high UA concentrations but not with normal UA concentrations.

### High UA increased AR expression via p38/MAPK pathway

**Fig. 2:**
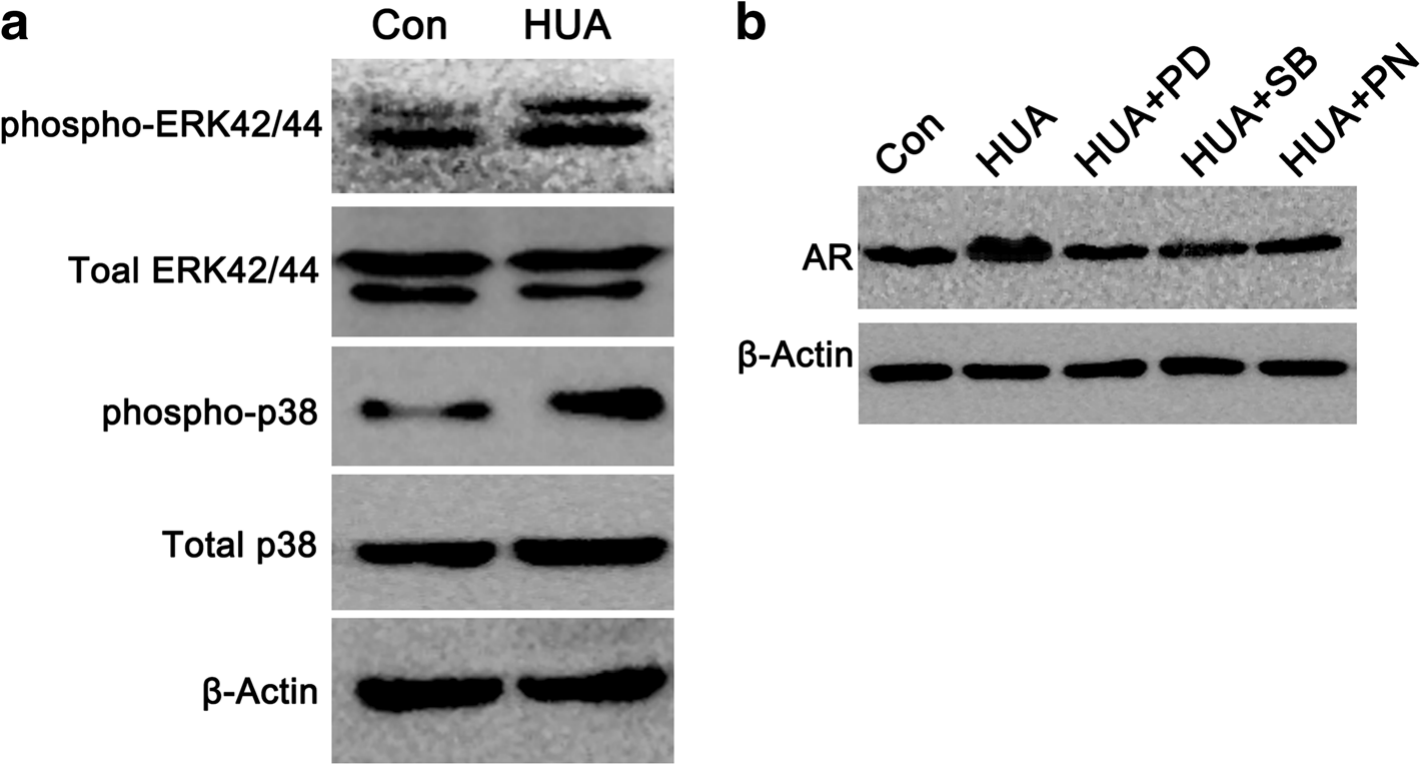
High UA increased AR expression via p38/MAPK pathway. Effect of UA on mitogen-activates protein kinase (MAPK) pathway activation (**a**). UA activated p38 and extracellular signal–regulated kinase (ERK) 44/42 MAPK pathway in HUVEC. Western blots shown are representative of four experiments for phosphorylated and total p38 and phosphorylated and total ERK44/42. Effect of co-stimulation of UA with MAPK inhibitors and probenecid on AR protein expression (B). UA-induced expression of AR (600 μM, 48 h) was blocked by inhibitors of p38 (SB203580, 5 μM), ERK42/44 (PD 98059, 10 μM), MAPK, and probenecid (PN)

In order to assay how UA trigger AR expression. P38 and extracellular signal–regulated kinase (ERK) 42/44 MAPK phosphorylation are involved in the UA-induced cell proliferation and activation in the UA-induced HUVEC [12]. Therefore we determined the effect of blocking p38 and ERK44/42 MAPK in UA-induced AR expression using specific inhibitors of the p38 (SB203580, 5 μM) and ERK44/42 (PD 98059, 10 μM) MAPK pathways respectively. Also, we used the organic anion transporter inhibitor, probenecid (Sigma-Aldrich, St. Louis, MO, USA), to block the uric acid transport into cells. We found AR expression increased when HUVECs were treated with high UA for 48 h, p38 and p-ERK42/44 were activated simultaneously [Fig. 2a]. When blocking p38 and MAPK by specific inhibitor SB203580 and PD 98059 respectively, or blocking the organic anion transporter that could transport uric acid into intracellular by probenecid, AR protein expression decreased [Fig. 2b].

### Increased AR expression enhances ROS production by activating NADPH oxidase

**Fig. 3:**
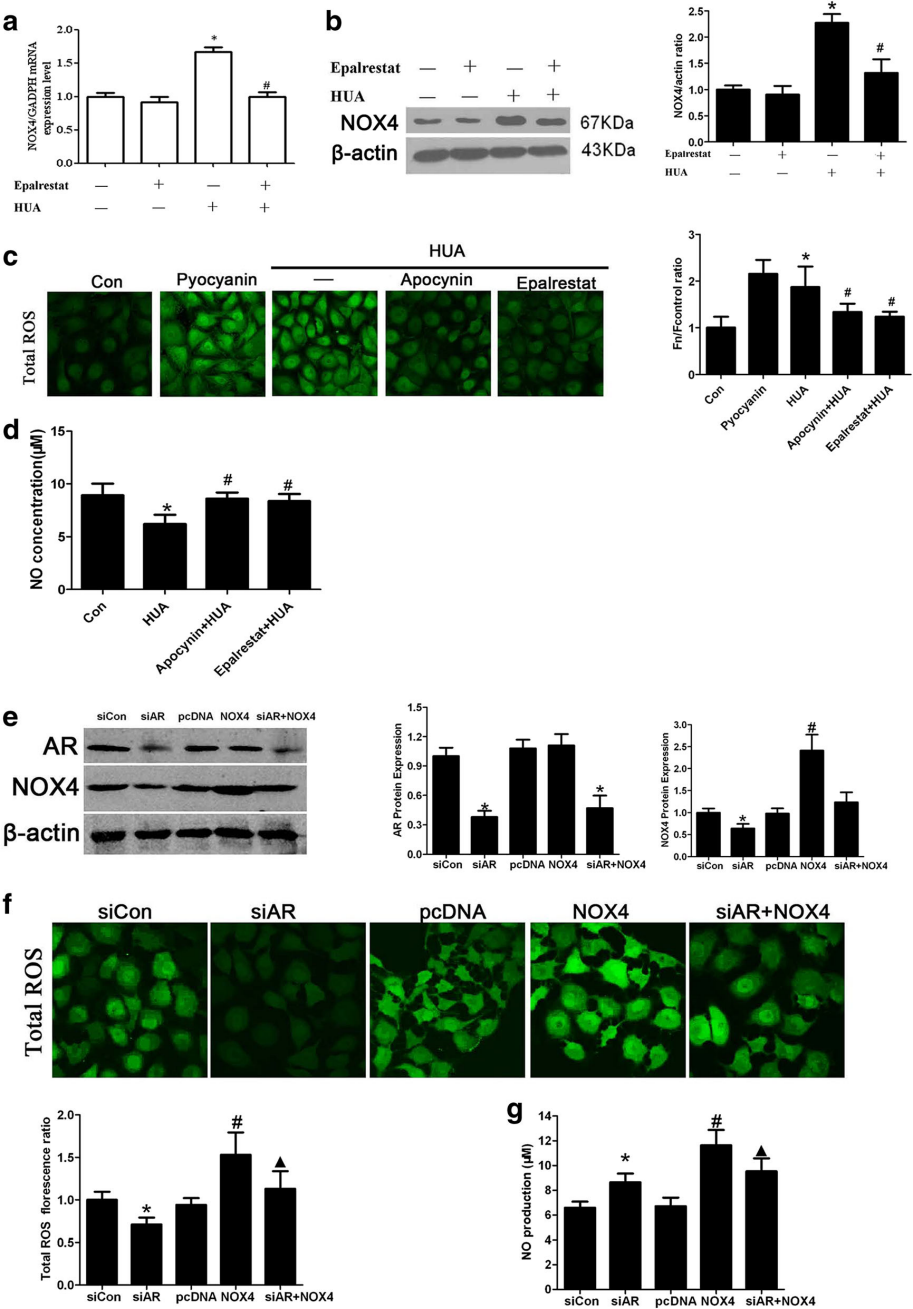
The AR inhibitor alleviated oxidative stress and impaired HUVECs by inhibiting NADPH oxidase activity. **a** and **b** Nox4 production was up-regulated in endothelial cells cultured in the presence of 600 μM UA for 24 h (*P < 0.05 vs. control, *n* = 6), but down-regulated in Epalrestat + HUA cells pretreated with epalrestat (0.1 μM) for 30 min, followed by incubation with UA (600 μM) for 24 h (^#^ *P <* 0.05 vs. HUA group, *n* = 6). **c** ROS production increased in endothelial cells incubated with UA (600 μM) for 24 h (*P < 0.05 vs. control, *n* = 6), was similar to that in cells treated with pyocyanin, decreased in endothelial cells when pretreated with apocynin/epalrestat (^#^ *P <* 0.05 vs. HUA group, *n* = 6), and was reduced significantly in cells pretreated with the AR inhibitor epalrestat (^†^ *P <* 0.05, Apocynin + HUA group vs. Epalrestat + HUA group, *n* = 6). **d** NO levels in supernatant in the Epalrestat + HUA group were enhanced compared to those in the high UA group after endothelial cells were pretreated with epalrestat for 30 min followed by UA (600 μM) for 24 h (*P < 0.05 vs. HUA group, *n* = 6). **e–g** Cells were transfected with siRNA or pcDNA3-NOX4, and treated with high uric acid for 24 h. AR siRNA knocked-down AR protein expression and downregulated NOX4 expression (*P < 0.05 vs. siCon group, *n* = 6). ROS production decreased and NO production increased (*P < 0.05 vs. siCon group, *n* = 6). However, overexpression of NOX4 did not affect AR. If cells were treated with AR RNAi and overexpressed NOX4, ROS production and NO concentration significantly decreased and increased, respectively, compared to the NOX4 overexpression group (▲*P <* 0.05, *n* = 6)

NOX4 is the main NOX as well as the main source of ROS in endothelial cells under oxidative stress [38–40]. NOX4 mRNA and protein expression levels increased in endothelial cells following challenge with high UA (Fig. 3a and b), but not NOX2, An additional file shows this in more detail [see Additional file 2]. However, when pretreated with the NOX inhibitor apocynin, ROS production induced by high UA levels was reduced (Fig. 3c). However, NOX4 expression was downregulated following pretreatment with the AR inhibitor epalrestat before high UA treatment, (Fig. 3a and b), suggesting that the enhanced AR expression induced by high UA activates NOX, thereby upregulating ROS expression and ultimately impairing endothelial cells. The AR inhibitor enhanced NO production compared with that in the high UA group (Fig. 3d), suggesting that inhibiting ROS production protected endothelial cells. However, when NOX4 was overexpressed in combination with AR knockdown, high UA treatment significantly decreased ROS production compared with that of cells overexpressing NOX4 alone. Furthermore, NO secretion concomitantly increased (Fig. 3e–g).

### High UA impaired endothelial cells by enhancing H_2_O_2_ production but inhibited other ROS components

**Fig. 4:**
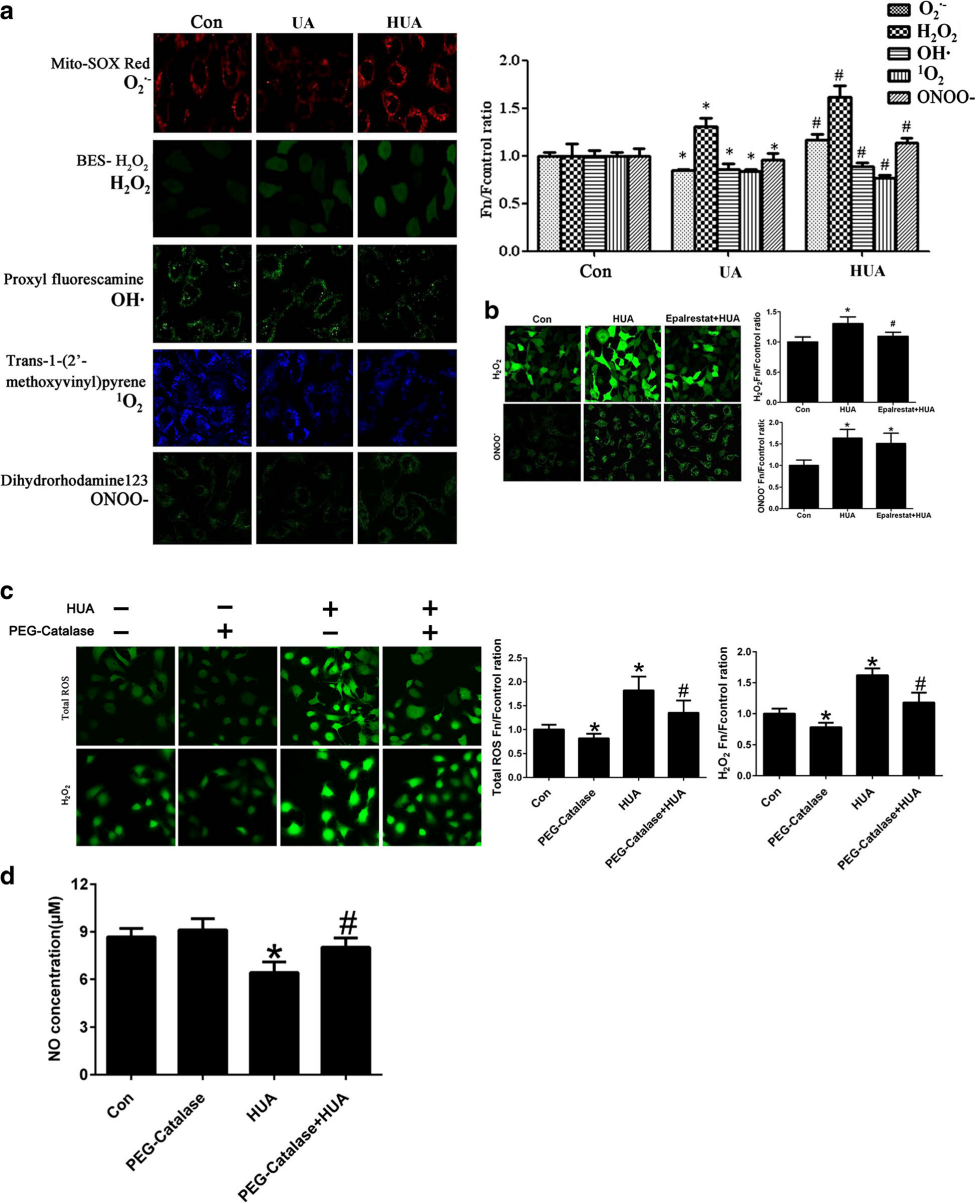
H_2_O_2_ production increased in endothelial cells treated with various concentrations of UA, and impaired endothelial cells were reduced after elimination of H_2_O_2_ (A) O_2_^•−^, ^1^O_2_, ·OH, and ONOO^−^ levels decreased in endothelial cells (*P < 0.05 vs. control, *n* = 6), but H_2_O_2_ was up-regulated in endothelial cells treated with 300 μM UA for 24 h (*P < 0.05 vs. control, *n* = 6). ^1^O_2_ and · OH levels were down-regulated in endothelial cells treated with 600 μM UA for 24 h (^#^ *P <* 0.05 vs. control, *n* = 6), whereas O_2_^•−^, H_2_O_2_, and ONOO^−^ levels were up-regulated in endothelial cells treated with 600 μM UA (^#^ *P <* 0.05 vs. control, *n* = 6). **b** H_2_O_2_ production decreased in epalrestat + HUA cells that were pretreated with epalrestat for 30 min followed by treatment with 600 μM UA for 24 h (*P < 0.05 vs. HUA group, *n* = 6), but ONOO^−^ levels in Epalrestat + HUA cells did not change compared to the HUA group. **c** Tntracellular H2O2 and total ROS levels were reduced by using PEG-catalase in the high UA cells, and NO production was enhanced in PEG-catalase + high UA cells pretreated with PEG-catalase for 30 min followed by treatment with 600 μM UA for 24 h (*P < 0.05 vs. HUA group, *n* = 6)

We then assessed the production of four ROS components after treatment with various UA concentrations. UA partially eliminated superoxide anion (O_2_^•−^), ^1^O_2_, and hydroxyl radical (·OH) production and subtly increased H_2_O_2_ production. However, high UA treatment increased O_2_^•−^ and H_2_O_2_ production but reduced ^1^O_2_ and · OH production (Fig. 4a).

After high UA treatment, O_2_^•−^ and H_2_O_2_ levels increased. Although O_2_^•−^ is highly dynamic, it soon became disproportionate to H_2_O_2_. Therefore, we inferred that H_2_O_2_ is the major ROS contributor to endothelial cell impairment induced by high UA treatment. H_2_O_2_ levels significantly decreased in endothelial cells pretreated with epalrestat followed by high UA treatment, but ONOO^−^ levels did not change (Fig. 4b). When PEG-catalase was applied to eliminate intracellular H_2_O_2_, total intracellular ROS levels were reduced, and NO levels increased compared with the high UA group that did not receive PEG-catalase treatment (Fig. 4c, d).

### PEG-catalase and AR inhibitor epalrestat reduce H_2_O_2_ production in hyperuricemic mice and effectively protect mouse endothelial cell function

**Fig. 5:**
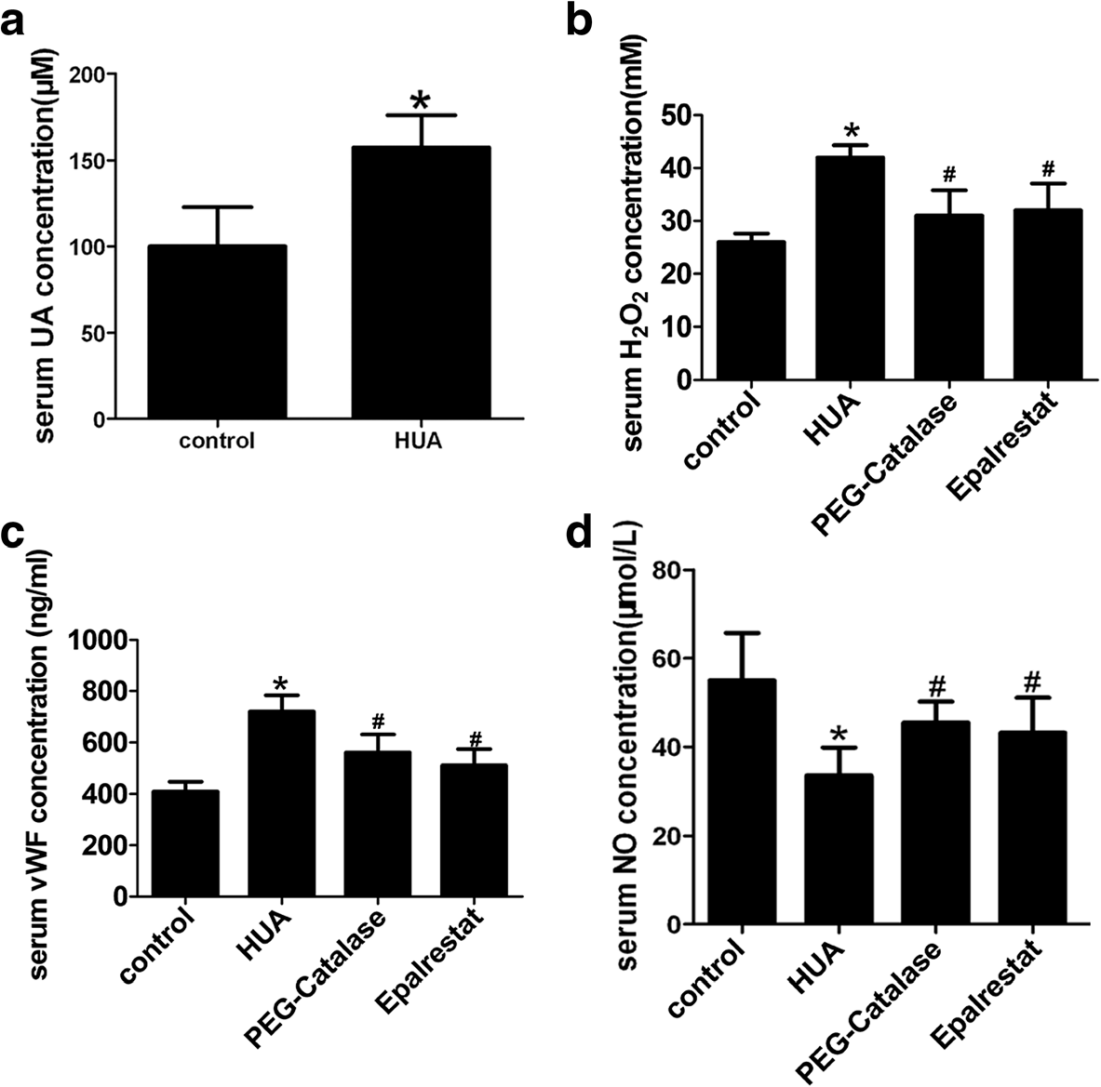
The AR inhibitor reduced H_2_O_2_ production and protected endothelial function. **a** After intraperitoneal injection with oxonic acid potassium salt and UA for 10 days, serum UA levels in wild-type C57BL/6 mice increased significantly (*P < 0.05 vs. control, *n* = 6), (**b**) Serum H_2_O_2_ levels increased in non-treated hyperuricemic mice (*P < 0.05 vs. control, *n* = 6). AfterPEG-catalase or epalrestat treatment, serum H_2_O_2_ levels declined significantly compared to the non-treated hyperuricemic group (^#^ *P <* 0.05 vs. HUA group, *n* = 6). **c** Serum vWF levels increased in the hyperuricemic mouse model (*P < 0.05 vs. control, *n* = 6). After treatment with PEG-catalase or epalrastat, serum vWF levels declined significantly compared to the non-treated hyperuricemic group (^#^ *P* < 0.05 vs. HUA group, *n* = 6). **d** NO levels decreased in the hyperuricemic model (*P < 0.05 vs. control, *n* = 6). After treatment with PEG- catalase or epalrastat, serum NO levels increased significantly compared to the non-treated hyperuricemic group (# *P* < 0.05 vs. HUA group, *n* = 6)

Elevated H_2_O_2_ can lead to vascular endothelial dysfunction [41]. H_2_O_2_ production increased in endothelial cells after high UA treatment in vitro (Fig. 3a). Upon endothelial dysfunction, cells release more vWF, which is a marker of endothelial dysfunction [42–44]. To estimate vascular endothelial function in vivo, we detected serum vWF concentrations. Serum H_2_O_2_ and vWF (Fig. 5b ~ d) levels increased in the hyperuricemic model, while the NO concentration decreased. We concluded that AR, O_2_^•−^, and H_2_O_2_ are the major contributors to impaired endothelial cell function. Inhibiting these could reduce the number of impaired endothelial cells induced by high UA treatment. After treatment with PEG-catalase or epalrestat, serum H_2_O_2_ (Fig. 5b) and vWF (Fig. 5c) levels decreased compared with those of the untreated group, while NO concentration increased (Fig. 5d). Based on the PEG-catalase treatment results, H_2_O_2_ may be the final ROS product responsible for endothelial cell impairment. Our data suggest that PEG-catalase and epalrestat decrease H_2_O_2_ production, thereby protecting endothelial function.

## Discussion

Uric acid (UA), which is generated in mammalian systems as an end product of purine metabolism, is the most abundant antioxidant in human plasma and possesses free radical scavenging properties. In humans and other higher primates, uric acid is the final compound of purines catabolism, but all other mammals converts uric acid to allantoin with enzyme uricase which is deficient in humans and other higher primates [2], and is the main reason why serum UA levels in adult males are 350 μmol/L, compared with the majority of mammals who have UA levels <30–60 mg/dl [45]. Evidence has demonstrated Western diet could elevate serum uric acid [46]. However, it may also act as a pro-oxidant under oxidative stress conditions. Markedly increased UA levels cause gout and nephrolithiasis [47], and high UA concentrations are also associated with an increased risk of developing cardiovascular disease (CVD), particularly hypertension, obesity/metabolic syndrome, and kidney disease [10, 11, 48–51]. Jia et al. verified hyperuricemia is related with the development of obesity/metabolic cardiomyopathy [46]. However, the role of UA in CVD pathogenesis is still debated. UA is one of the most important antioxidants in body fluids and effectively eliminates ROS [52]. Other risk factors exist in CVD patients in addition to the superoxide generation that accompanies UA production by xanthine oxidoreductase [53]. Whether UA is a causative risk factor or plays a protective role with respect to its antioxidant properties is not known [54, 55]. The mechanism (s) by which UA acts as a “double-edged sword” remain to be determined.

Mounting evidence indicates that hyperuricemia induces heart and kidney injury by promoting free radical generation and subsequent endothelial dysfunction [56], which are regulated by NO bioavailability and activity changes [57, 58]. UA possesses the potential to downregulate NO production and induce endothelial injury through at least three mechanisms, namely modulating the eNOS phosphorylation status, potentiating arginase activity, and increasing intracellular superoxide levels [59]. Because UA is a powerful free radical scavenger, we first investigated changes in levels of the major free radicals in the presence of different UA concentrations in the endothelium. The four major cellular ROS components are Mito-O_2_^•-^, ·OH, ^1^O_2_, and H_2_O_2_, all of which can be interconverted [38, 60–63]. Here, we observed that under normal UA concentrations (300 μM), UA suppresses O_2_^•-^, ·OH, and ^1^O_2_ release and slightly increases H_2_O_2_. The slight increase in H_2_O_2_ levels (Fig. 4a) may not be harmful because low H_2_O_2_ concentrations can protect endothelial function [64, 65] and affect vasodilation [66]. When the UA concentration was increased to 600 μM, ^1^O_2_ and · OH release remained suppressed, whereas O_2_^•-^ and H_2_O_2_ levels significantly increased. Because H_2_O_2_ is generated from reduced O_2_^•-^ by superoxide dismutase (SOD), high UA levels likely did not suppress O_2_^•-^ release but rather stimulated O_2_^•-^ generation. This result is similar to previous reports [3, 67]. Although it is not a free radical, we also measured ONOO^−^ levels, ^−^ which is a highly toxic molecule that can cause harmful effects. Under normal conditions, the ONOO^−^ level remains low due to the low level of O_2_^•-^ generation. However, if O_2_^•-^ generation is enhanced, ONOO^−^ production also increases [68–70]. Because UA can scavenge ONOO^−^, ONOO^−^ levels decreased in the presence of normal UA concentrations. The increase in ONOO^−^ levels resulting from high UA treatment was likely due to elevated O_2_^•-^ levels. These results suggest that enhanced O_2_^•-^ generation plays a central role in high UA-induced endothelial dysfunction.

**Fig. 6:**
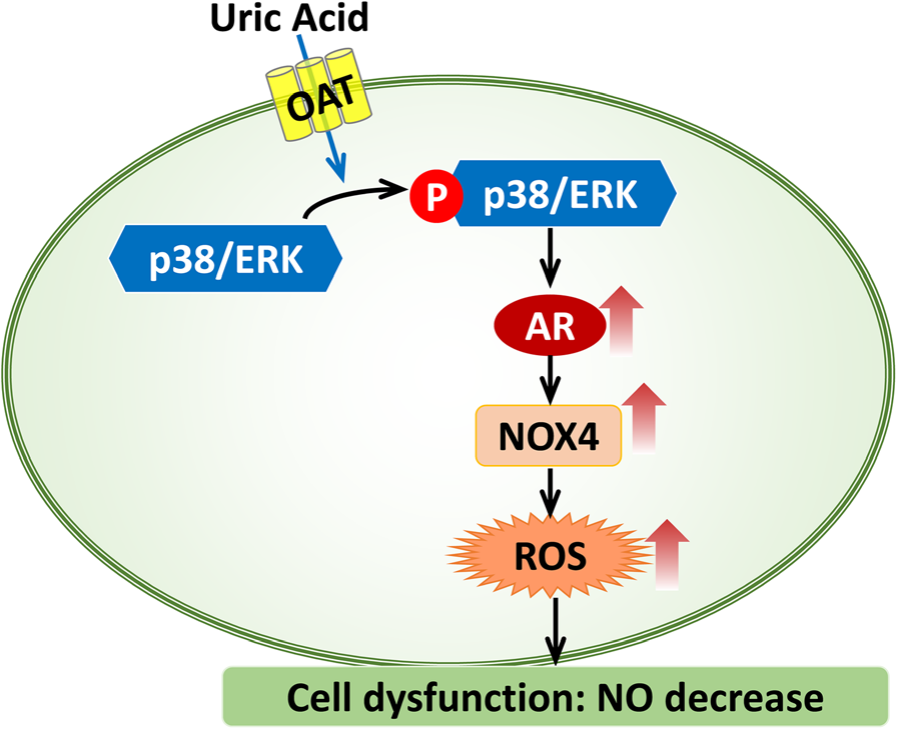
The mechanism of uric acid inducing endothelial dysfunction. OAT: organic anion transporter. AR: aldose reductase, NOX4: nicotinamide adenine dinucleotide phosphate oxidase 4, ROS: reactive oxygen species, NO: nitric oxide

Our previous study demonstrated high concentration uric acid could induce AR mRNA and protein expression level of HUVECs [24]. Johnson et al. reported that p38 and ERK44/42 MAPK pathways are activation in rat VSMC incubated with uric acid [71, 72]. Later, they confirmed the activation of p38 and ERK44/42 MAPK pathways were involved in HVSMC and endothelial cells treated with uric acid [12]. In order to try to clarify whether high UA mediate AR expression increase via above signal pathway. We observed AR expression could be repressed when using p38 or MAPK inhibitor respectively, or the organic anion transporter blocker probenecid. It implied that high UA might mediated AR expression via p38/MAPK pathway (Fig. 6). Researchers demonstrated p38 could activate osmotic response element-binding protein/tonicity-responsive enhancer-binding protein (OREBP/TonEBP), transcriptional factors, which bind AR promoter then induce its expression [73]. We also measured the activity of the two protein. Yet in the high concentration uric acid environment, the expression or the activity of OREBP/TonEBP did not increase (data was not shown). The mechanism of the elevated AR expression and activity induced by uric acid does not depend on the p38-OREBP/TonEBP. Nishikawa et al. considered that ROS production also can activated AR expression [74]. We observed that ROS produced at early time but AR expression obviously changed at 24 h. Our previous study proved high concentration of uric acid caused abnormal sodium-calcium exchanger of mitochondria then induced the ROS production [14], which will further increase AR expression and these may be locked into a destructive cycle. There might be other pathway mediate ROS production in the high concentration of uric acid.

When an AR inhibitor was added to the high UA-treated HUVECs, total ROS levels significantly decreased, and NO levels recovered. AR-induced ROS production is associated with NADPH [32, 33]; our results also suggested the involvement of NOX4 activation, but not NOX2 (Additional file 1). Upon blocking NOX4 with apocynin, ROS levels decreased, and NO levels recovered. When NOX4 was overexpressed In AR-knockdown HUVECs overexpressing NOX4 treated with high UA, ROS production and NO levels were the inverse of those resulting from NOX4 overexpression alone. Additionally, the AR inhibitor epalrestat affected H_2_O_2_ but not ONOO^−^ levels and increased NO levels. These results confirm that AR activation plays an important role in high UA-stimulated HUVECs.

The above results suggest that the high UA-induced increased O_2_^•-^ generation was associated with the switch of UA functioning as an antioxidant to a pro-oxidant in vitro. Because O_2_^•-^ is catalyzed to H_2_O_2_ by SOD in vivo, H_2_O_2_ is likely the major contributor to endothelial dysfunction. Additionally, serum UA levels correlate with plasma H_2_O_2_ in preeclampsia [75]. Therefore, blocking AR, reducing H_2_O_2_, or decreasing O_2_^•-^ would protect the endothelium from high UA-induced injury. In this study, epalrestat and PEG-catalase recovered NO secretion levels and decreased vWF levels.

Previously, we measured blood pressure in hyperuricemic wild-type mice and reported no difference between the two groups (data not shown). Endothelial dysfunction resulting from hyperuricemia would not impact blood pressure in the two-week model. However, if the duration of exposure to high UA concentrations was extended, the accumulation of endothelial dysfunction would cause artery dysfunction and dysarteriotony. These data are similar to the report by Johnson et al. [51, 76].

## Conclusion

Our study confirmed that the levels of ROS components changed in HUVECs cultured in media containing different UA concentrations. In particular, H_2_O_2_ significantly increased in the high UA group compared with the control group. The elevated ROS levels were reversed when AR was inhibited in vivo or in vitro. Thus, the pro-oxidant activity of UA when present at high concentrations likely plays an important role in endothelial dysfunction via AR.

## Additional files

Additional file 1: ROS and NO production induced by uric acid. (PDF 104 kb)
Additional file 2: NOX2 and NOX4 expression induced by high uric acid. (PDF 107 kb)

## Abbreviations

^1^O_2_: Singlet oxygen
AR: Aldose reductase
H_2_O_2_: Hydrogen peroxide
HUVEC: Human umbilical vein endothelial cells
NO: Nitric oxide
NOX: NADPH oxidase
O_2_^•-^: Superoxide anion
OH: Hydroxyl radical
ONOO^−^: Peroxynitrite
ROS: Reactive oxygen species
SOD: Superoxide dismutase
UA: Uric acid
VWF: von Willebrand factor
