# Supplementary material for: Aldose reductase mediates endothelial cell dysfunction induced by high uric acid concentrations
Source: Cell Commun Signal. 2017 Jan 5;15:3. doi: 10.1186/s12964-016-0158-6 (PMC5217275; doi:10.1186/s12964-016-0158-6)
Supplement: Additional file 1: — ROS and NO production induced by uric acid. (PDF 104 kb) [file 12964_2016_158_MOESM1_ESM.pdf]

Additional file 1：ROS and NO production induced by uric acid

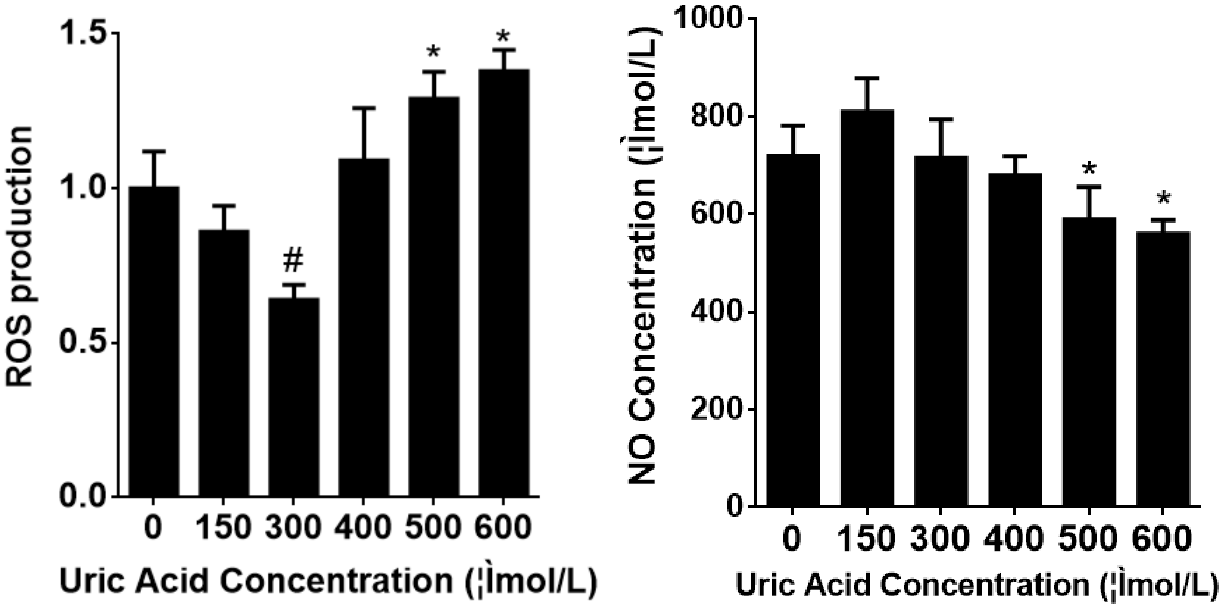

ROS production and NO concentration of HUVEC-c cell when cultured with different uric acid concentration (0, 150, 300, 400, 500 and 600μmol/L). At the point of 500μmol/L uric acid, ROS production had a significantly elevation when compared with control (0μmol/L), #,*p<0.05, and the NO concentration decreased significantly as well, *P<0.05.
