# Supplementary material for: Aldose reductase mediates endothelial cell dysfunction induced by high uric acid concentrations
Source: Cell Commun Signal. 2017 Jan 5;15:3. doi: 10.1186/s12964-016-0158-6 (PMC5217275; doi:10.1186/s12964-016-0158-6)
Supplement: Additional file 2: — NOX2 and NOX4 expression induced by high uric acid. (PDF 107 kb) [file 12964_2016_158_MOESM2_ESM.pdf]

Additional file 2： NOX2 and NOX4 expression induced by high uric acid

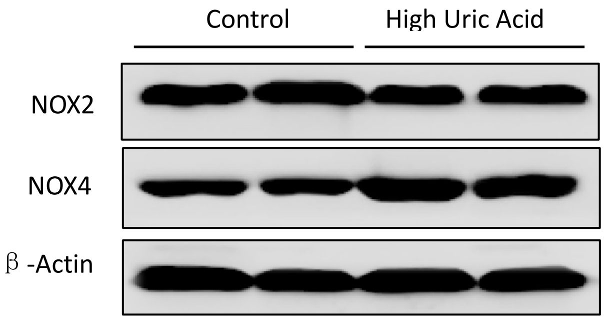

NOX2 and NOX4 protein level in endothelial cells incubated with high concentration uric acid (600μM) assayed by western blot. High concentration uric acid could not increase NOX2 protein expression level, but NOX4.
